# Supplementary material for: Synthesis and Characterization of a Novel Resveratrol Xylobioside Obtained Using a Mutagenic Variant of a GH10 Endoxylanase
Source: Antioxidants (Basel). 2022 Dec 30;12(1):85. doi: 10.3390/antiox12010085 (PMC9855058; doi:10.3390/antiox12010085)
Supplement: Supplementary file 1 [file antioxidants-12-00085-s001.zip › antioxidants-2095067-supplementary.pdf]

Supplementary Material

Synthesis and Characterization of a Novel Resveratrol Xylobioside Obtained Using a Mutagenic Variant of a GH10 Endoxylanase

Ana Pozo-Rodríguez [1], Juan A. Méndez-Líter [1], Rocío García-Villalba [2], David Beltrán [2], Eva Calviño [3,4], Andrés G. Santana [5], Laura I. de Eugenio [1], Francisco Javier Cañada [3,4], Alicia Prieto [1], Jorge Barriuso [1], Francisco A. Tomás-Barberán [2] and María Jesús Martínez [1,*]

[1] Department of Microbial and Plant Biotechnology, Centro de Investigaciones Biológicas Margarita Salas, Spanish National Research Council (CIB, CSIC), C/Ramiro de Maeztu 9, 28040 Madrid, Spain
[2] Department of Quality, Safety and Bioactivity of Plant Foods, Centro de Edafología y Biología Aplicada del Segura, Spanish National Research Council (CEBAS, CSIC), Espinardo, 30100 Murcia, Spain
[3] Department of Structural and Chemical Biology, Centro de Investigaciones Biológicas Margarita Salas, Spanish National Research Council (CIB, CSIC), C/Ramiro de Maeztu 9, 28040 Madrid, Spain
[4] CIBER de Enfermedades Respiratorias (CIBERES), Avda. Monforte de Lemos 3-5, 28029 Madrid, Spain
[5] Department of Chemistry of Natural and Synthetic Bioactive Products, Instituto de Productos Naturales y Agrobiología, Spanish National Research Council (IPNA, CSIC), Avda. Astrofísico Francisco Sánchez 3, 38206 San Cristóbal de La Laguna, Spain
* Correspondence: mjmartinez@cib.csic.es

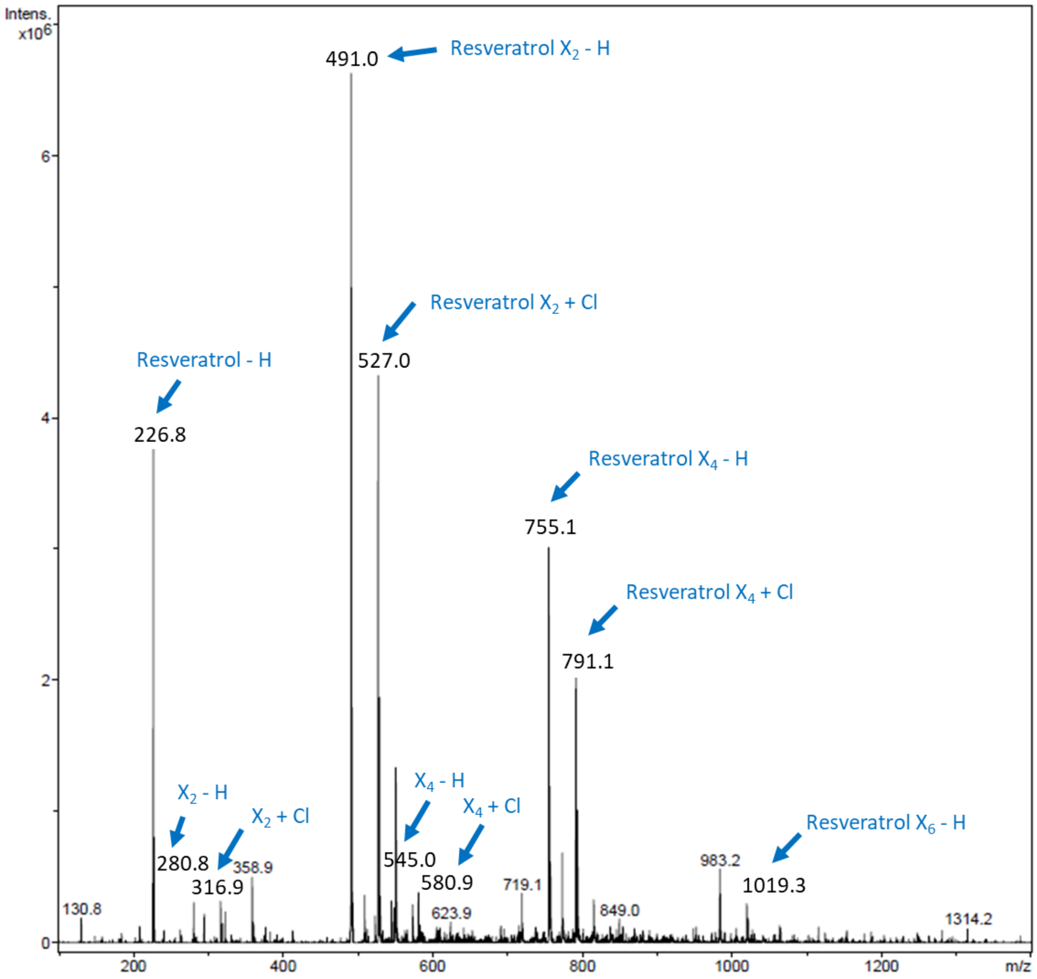

Figure S1: ESI-MS spectrum (negative mode) of the standard resveratrol glycosylation reaction catalyzed by rXynSOS-E236G glycosynthase using X_2F as the donor. The m/z of ions corresponding to the H and Cl adducts of resveratrol glycosides, xylooooligosacharides and non-glycosylated resveratrol are indicated in blue arrows.

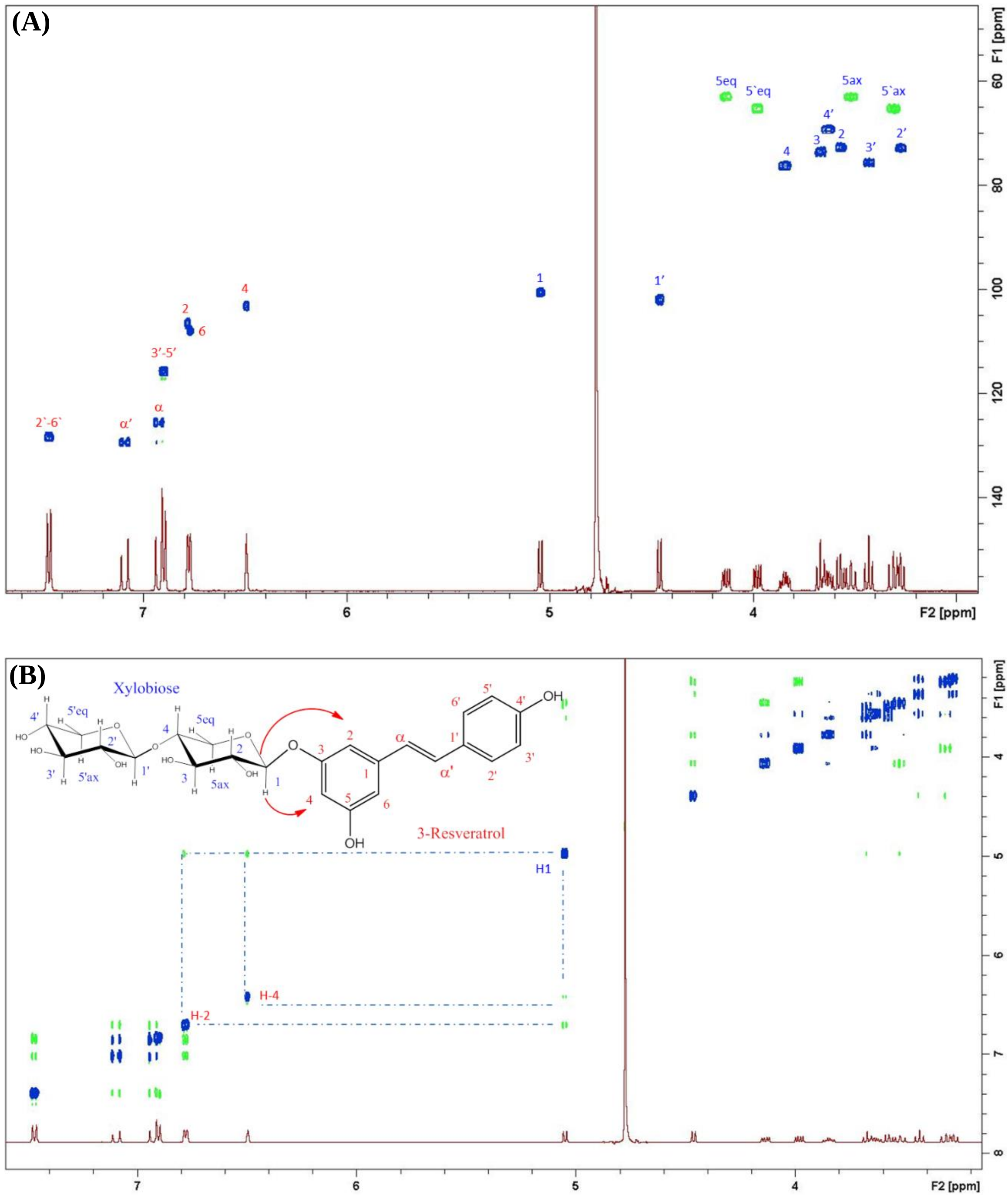

Figure S2: NMR spectra of glycoside 1 dissolved in deuterated water that corresponds to 3-*O*-β-D-xylobiosyl resveratrol. (A) Superimposition of ^{1}H zg (cherry) and ^{1}H-^{13}C HSQC (blue/green) spectra and assignation, xylobiose (blue), resveratrol (red). (B) Superimposition of ROESY (blue/green) and ^{1}H zg (cherry) spectra with highlighted ROE correlation between ^{1}H 1 of xylobiose and ^{1}H 2 and ^{1}H 4 of resveratrol (blue lines).

Table S1. Chemical shifts for 3-*O*-β-D-xylobiosyl resveratrol dissolved in deuterated water. Chemical shifts were referenced to the residual water signal set at 4.77 ppm at 298 K.

3-Resveratrol	^{1}H (ppm)	^{13}C (ppm)
**1**		140*
**2**	6.78	106.29
**3**		157.9*
**4**	6.49	103
**5**		156.8*
**6**	6.77	107.87
**α**	6.92	125.4
**α'**	7.1	129.3
**1'**		129.4*
**2' (6')**	7.46	128.3
**3' (5')**	6.89	115.0
**4'**		155.4*

*Assignments derived from HMBC spectrum

Xylobiose	^{1}H (ppm)	^{13}C (ppm)
1	5.05	100.5
2	3.57	72.61
3	3.66	73.63
4	3.84	76.06
5 ax	3.52	62.94
5 eq	4.13	62.94
1'	4.46	104.5
2'	3.27	75.3
3'	3.43	78.1
4'	3.63	71.7
5'ax	3.3	67.7
5'eq	3.98	67.6

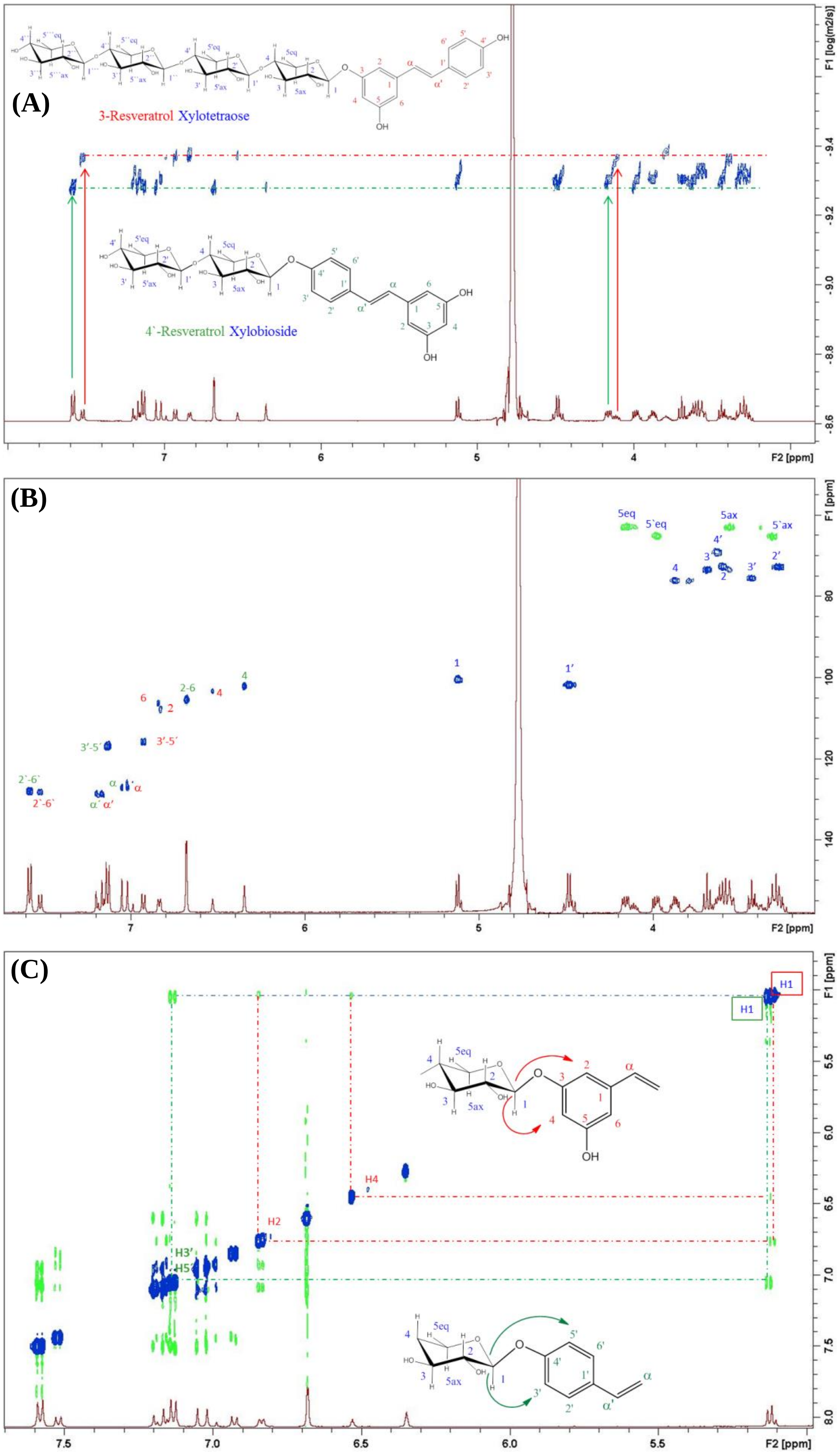

Figure S3: NMR spectra of glycoside 2 dissolved in deuterated water that corresponds to a mixture of 4'-*O*-β-D-xylobiosyl resveratrol and 3-*O*-β-D-xylotetraosyl resveratrol in a 3:1 ratio. (A) . Superimposition of DOSY (blue) and 1D-1H (cherry) spectra with different diffusion coefficient values of 4'-*O*-β-D-xylobiosyl resveratrol (green lines) and 3-*O*-β-D-xylotetraosyl resveratrol (red lines). (B) Superimposition of 1D-1H (cherry) and 1H-13C HSQC (blue/green) spectra and assignation, xylobiose (blue), 3-resveratrol (red) and 4'-resveratrol (green). (C) Superimposition of ROESY (blue/green) and 1D-1H (cherry) spectra with highlighted ROESY correlation between H1 of xylobiose moiety and H3'and H5' of resveratrol moiety in the case of 4'-*O*-β-D-xylobiosyl resveratrol (green lines) and between H1 of xylotetraose moiety and H2and H4 of resveratrol moiety for 3-*O*-β-D-xylotetraosyl resveratrol (red lines).

Table S2. Chemical shifts for 4'-*O*-β-D-xylobiosyl resveratrol (A) and 3-*O*-β-D-xylotetraosyl resveratrol (B) dissolved in deuterated water. Chemical shifts were referenced to the residual water signal set at 4.77 ppm at 298 K.

(A) 4'-*O*-β-D-xylobiosyl resveratrol

4'-Resveratrol	^{1}H	^{13}C
**1**		126.7*
**2 /6**	6.7	105.4
**4**	6.34	102.6
**3(5)**		105.2
**α**	7.03	127.1
**α'**	7.18	128.9
**1'**		131.8*
**2' (6')**	7.58	128.0
**3' (5')**	7.1	116.9
**4'**		155.8*

*Assignments derived from HMBC spectrum

Xylobiose	^{1}H	^{13}C
1	5.12	100.5
2	3.60	72.7
3	3.69	73.5
4	3.87	76.1
5 ax	3.56	62.8
5 eq	4.15	62.8
1'	4.48	101.7
2'	3.29	72.7
3'	3.43	75.5
4'	3.63	69.0
5'ax	3.31	65.1
5'eq	3.98	65.1

(B) 3-*O*-β-D-xylotetraosyl resveratrol

3-Resveratrol	^{1}H	^{13}C
**1**		
**2**	6.8	107.6
**4**	6.5	103.2
**6**	6.8	106.4
**α**	6.98	125.7
**α'**	7.18	128.9
**1'**		131.8*
**2' (6')**	7.52	128.1
**3' (5')**	6.9	115.9
**4'**		

*Assignments derived from HMBC spectrum

Xylotetraose	^{1}H	^{13}C
1	5.11	100.5
2	3.56	73.3
3	3.69	73.5
4 4' 4"	3.87/3.79	76.1
5 5' 5" ax	3.56/3,38	62.8
5 5' 5" eq	4.16-4.10	62.9
1'1" 1"'	4.5-4.45	101.7
2' 2" 2"'	3.26-3.29	72.7
3' 3" 3"'	3.43-3.42	75.5
4"'	3.63	69.0
5"'ax	3.31	65.1
5"'eq	3.96	65.1

Data S1: Equations for maximum production and maximum conversion (%) for the major resveratrol xylobioside (3-*O*-β-D-xylobiosyl resveratrol)

3-*O*-β-D-xylobiosyl resveratrol maximum production was adjusted with the following model equating:

Production (g/L) = 0.308114 + 0.012853 * [Resveratrol] – 0.004821 * [X_2F] + 0.033544 * [rXynSOS-E236G] + 0.172893 * Time + 0.004792 * [Resveratrol] * [X_2F] + 0.066433 * [Resveratrol] * [rXynSOS-E236G] + 0.011107 * [Resveratrol] * Time – 0.006159 * [X_2F] * [rXynSOS-E236G] – 0.004073 * [X_2F] * Time – 0.069273 * [rXynSOS-E236G] * Time

3-*O*-β-D-xylobiosyl resveratrol maximum conversion was adjusted with the following model equating:

Conversion (%) = 15.92844 – 0.241641 * [Resveratrol] + 0.056521 * [X_2F] + 6.54882 * [rXynSOS-E236G] + 2.38029 * Time + 0.089139 * [Resveratrol] * [X_2F] + 0.849929 * [Resveratrol] * [rXynSOS-E236G] + 0.317365 * [Resveratrol] * Time – 0.102658 * [X_2F] * [rXynSOS-E236G] – 0.067877 * [X_2F] * Time – 1.15454 * [rXynSOS-E236G] * Time – 0.278498 * $[\text{Resveratrol}]^2$ – 0.007765 *$[X_2F]^2$ – 1.15589 * $[\text{rXynSOS-E236G}]^2$ – 0.104417 * Time^2
